# Arrests and the Opioid Epidemic: An Investigation into the Spatial and Social Network Spillover of Opioid Overdoses in Chicago

**DOI:** 10.1007/s10940-025-09612-y

**Published:** 2025-05-31

**Authors:** Megan Evans, Corina Graif, Anna Newell

**Affiliations:** 1 Max Planck Institute for Demographic Research, Konrad-Zuse-Str. 1, 18057 Rostock, Germany; 2 Max Planck - University of Helsinki Center for Social Inequalities in Population Health, Rostock, Germany; 3 Pennsylania State University, University Park, PA, USA

**Keywords:** Opioid Epidemic, Drug Policy, Spatial-Network Spillovers

## Abstract

**Objectives:**

This study investigates the role of criminal justice intervention practices, i.e., opioid arrests, in effectively preventing or increasing opioid overdoses, paying particular attention to whether arrests in spatially proximate or socially connected communities lead to the displacement or prevention of opioid overdoses in a local community.

**Methods:**

Combining data from the Cook County medical examiner, emergency medical services information, arrest reports, and commuting network statistics for Chicago’s 77 community areas between 2016 and 2019, this study uses fixed effects spatial autoregressive models with spatial lags to explain community-level opioid overdose rates.

**Results:**

We find evidence for the diffusion and displacement of overdose risk as well as the diffusion of overdose-reducing benefits. Findings suggest complex spatial and social spillover mechanisms that both diffuse and prevent opioid overdoses, dependent on the type of opioid-related crime and overdose rate investigated.

**Conclusions:**

These results have important implications for understanding the effectiveness of criminal justice policies in their goal of preventing opioid-related crime and overdoses and provide insights for designing more appropriate and effective policy responses to address substance use and illicit drug markets.

## Introduction

A steep climb in overdose deaths from synthetic opioids such as tramadol and fentanyl in 2017 led the U.S. Department of Health and Human Services to declare the opioid epidemic a national public health emergency (Carroll et al. 2018). Over seven hundred thousand people died from an opioid-related overdose between 1999 and 2022 (CDC 2024), contributing to an unprecedented stagnation in U.S. life expectancy (Harper et al. 2021; Case and Deaton 2015). Public health officials and policy makers responded to the opioid crisis with multiple harm reduction interventions and regulations with the goal of reducing fatal overdoses from the overprescription of opioids and the use of illicit opioids. Nonetheless, criminal justice approaches have continued to remain a dominant strategy across the U.S. to combat the opioid epidemic (Caulkins et al. 2021; Donnelly et al. 2021, 2022).

Law enforcement activities leading to drug-related arrests are intended to function as formal social control mechanisms to regulate human behavior and prevent further crime and drug use (Braga et al. 2017; Sampson 1986), and in the case of opioid arrests, prevent further opioid misuse and overdose (Donnelly et al. 2021; Mazerolle et al. 2007; 2020). Arresting individuals in possession of opioids or individuals manufacturing and distributing opioids can both physically remove opioids from a community by disrupting drug supply networks as well as socially deter the misuse of opioids (Eggins et al. 2020; Holland et al. 2023; Mazerolle et al. 2007; 2020). However, law enforcement interventions have been shown to also produce unintended, adverse consequences on individuals and communities (Caulkins et al. 2021; Cooper et al. 2005; Kirk and Wakefield 2018).

As is the case with any social program aimed at crime prevention or harm reduction, it is imperative to understand the reality of the consequences, not the ideal of it (McDonald et al. 2024). A growing body of work investigating the relationship between the criminal justice system and the opioid epidemic shows that individuals who experience an opioid-related incarceration have an increased likelihood of overdosing upon release (Lim et al. 2012; Victor et al. 2021; Zhang et al. 2022). These effects impact not just individuals but entire communities as well. For example, police drug seizures of illicit opioids in a local community in Indiana during 2020–21 increased fatal overdoses locally and in geographically proximate areas (Ray et al. 2023), suggesting a diffusion of crime and health risk from the location of the seizure into geographically proximate spaces (Papachristos and Bastomski 2018; Sampson and Morenoff 2004; Tucker et al. 2012). While this growing body of research is helping shed light on the complex role the criminal justice system plays in the opioid epidemic, it is unclear how criminal justice sanctions in the form of opioid-related arrests influence opioid overdose rates and whether arrests can displace opioid risk into the broader community and city at large.

We propose that the diffusion of opioid overdose risk is related at least in part to human mobility between places. In this study, we thus aim to get closer to understanding diffusion mechanisms by modeling population commuting flows between places as conduits for transmitting opioid overdose risk across space. We go beyond the standard modeling of geographic proximity to measure risk spillovers and, instead, we examine risk transmission between communities that are socially connected through spatial networks based on commuting flows. These networks may help explain spatial spillovers but, more importantly, they are not constrained by proximity and can instead identify social influences across any geographic distance, small or large. Understanding the spatial networks of transmission that link arrests in one community to overdose risk in another is important for several key reasons. If studies examine the effects of law enforcement activities only locally or in nearby areas, they can miss important ripple effects on connected communities across the entire city. Indeed, a growing body of work has shown that neighborhoods are not closed systems (Graif et al. 2014, 2017; Sampson 2012). Not only are neighborhoods spatially interdependent, where criminogenic and health-related influences spillover into spatially proximate communities (Anselin 2000; Matthews and Yang 2013), but they are also socially connected via co-offending (Schaefer 2012), gang conflict (Papachristos et al. 2013), and the daily flow of individuals across the city as they conduct routine activities and commute for work (Boivin and D’Elia 2017; Boivin and Felson 2018; Browning et al. 2017; Evans et al. 2023; Felson and Boivin 2015; Graif et al. 2017; Newmyer et al. 2022; Wang et al. 2016). Crime and population health patterns are better explained when accounting for spillovers both spatially and socially (Anselin 2000; Baller et al. 2001; Evans et al. 2023; Graif et al. 2017; Levy et al. 2020; Newmyer et al. 2022; Sampson et al. 2002; Taylor 2015).

This study thus investigates the role of criminal justice intervention practices, i.e., opioid-related arrests, in preventing or increasing opioid overdoses, paying particular attention to whether arrests in spatially proximate and, importantly, also in socially connected communities lead to the displacement or prevention of opioid overdoses in a local community. Moreover, recognizing the multidimensional nature of crime prevention programs and spillover processes (McDonald et al. 2024; Papachristos and Bastomski 2018; Telep et al. 2014), this study assesses heterogeneity in the relationship between arrests and opioid overdoses by examining variation between: 1) *fatal* overdoses as reported by the Cook County Medical Examiner (CCME) versus *all* overdoses (fatal or not) responded to by emergency medical services (EMS), 2) arrests for *possession* versus arrests for *manufacturing and distribution*, and 3) arrests for *heroin* versus arrests for *synthetic narcotics*.^1^ Using the case of Chicago, we combine multiple sources of data, including reports from the Cook County medical examiner, emergency medical services information, arrest reports, and commuting statistics for Chicago’s 77 community areas between 2016 and 2019. This study uses fixed effects spatial autoregressive models with spatial lags to estimate community-level opioid overdose rates. We capture spatial spillovers from communities that share physical borders with one another and capture social spillovers from communities that are connected via daily commuting patterns. The results have important implications for understanding the effectiveness of criminal justice approaches in their goal of preventing opioid-related crime and overdoses. Moreover, the findings can provide insights when designing more appropriate and effective policy responses to address substance use and illicit drug markets.

## Theoretical and Empirical Background

### The Opioid Crisis in Chicago

Chicago provides a unique context for assessing the relationship between opioid-related arrests and opioid-related overdoses. For one, throughout the opioid epidemic Chicago has maintained a higher opioid-related overdose rate than the national average (Rushovich et al. 2022). For example, in 2015 the average number of deaths per 100,000 people in Chicago was 11.3, compared to 10.4 nationally and by 2017 the rates increased to 22.6 for Chicago compared to 14.9 nationally (Rushovich et al. 2022). While national fentanyl-related mortality rates began to increase in 2010, with a sharp increase in 2015, Chicago experienced upticks in fentanyl-related deaths as early as 2006 (Friedman and Shover 2023; Schumann et al. 2008). By 2016 over a half of the opioid overdose deaths were fentanyl related, a number that further increased to 74% by 2019.^2^ Overall, a total of 4,681opioid-related overdose fatalities occurred in Cook County, IL between 2016 and 2019^3^ (see also Knoebel and Kim 2023; Chicago Department of Public Health 2024).

As Chicago experienced trends of the overdose crisis earlier and with a higher magnitude than other regions in the U.S., the city has been at the forefront of introducing innovative harm reduction practices to address opioid-related harm, such as mobile medical units, naloxone distribution, and take-home naloxone programs (Eswaran et al. 2020; Hawk et al. 2015; Messmer et al. 2023). The first naloxone distribution program in the U.S. was started in Chicago in 1996 as a van-based harm reduction program called the Chicago Recovery Alliance (Hawk et al. 2015). In the decade following the creation of this program, the Chicago Recovery Alliance distributed naloxone to over ten thousand individuals and reversed over one thousand overdoses (Bivens 2019; Maxwell et al. 2006). The Chicago Recovery Alliance has since established six stationary sites and sixteen mobile sites (Bivens 2019).^4^ Thus, Chicago presents a unique case to investigate the relationship between law enforcement and the opioid epidemic given the magnitude of the opioid crisis and the existing harm reduction programs already in place in the city.

### Law Enforcement and the Opioid Epidemic: Local Effects

Law enforcement activities and the implicit threat of criminal justice sanctions have been traditionally used as formal control mechanisms in a community to regulate crime and deviant behavior, including illicit drug use (Braga et al. 2017; Sampson 1986). A community’s opioid-related arrest rate represents how law enforcement responds to substance use as well as the visibility of substance use to authorities. By arresting individuals in possession of, or manufacturing and distributing opioids, incarceration is intended to prevent substance use and overdoses. Incarceration will temporarily prevent substance use among the individuals arrested but also potentially inhibit access to opioids in the local illicit market by disrupting drug supply and information networks (Eggins et al. 2020). It can also deter drug use by impacting norms and attitudes about opioid use as individuals and peers hope to avoid future involvement in the criminal justice system. While the evidence in support of deterrence as a result of drug law enforcement activities is mixed, with some literature arguing that law enforcement discourages drug use and other literature arguing that it simply displaces it (Holland et al. 2023; Mazerolle et al. 2007, 2020), the traditional understanding of arrests as a crime control mechanism suggests the following hypothesis:

- Local health benefits hypothesis 1a: More local arrests would contribute to fewer opioid overdoses locally (deaths and EMS).

However, the effectiveness of police crackdowns and criminal justice contact is not always straightforward, and in fact, can backfire. Research shows that arrests for misdemeanors, including drug use in general and opioid misuse in particular, often has the opposite, unintended effect of increasing drug use and overdoses (Bohnert et al. 2011b; Krawczyk et al. 2020; Lim et al. 2012; Victor et al. 2021; Zhang et al. 2022). The criminalization of drugs and opioid misuse alongside with increasing police activity can deter individuals from seeking harm reduction and healthcare services if they become fearful of police involvement and subsequent arrest (Cooper et al. 2012; Ostrach et al. 2022). Additionally, at the individual level those who are arrested and placed in the criminal justice system have an increased likelihood of relapse and overdose upon release (Kopak et al. 2018; Krawczyk et al. 2020; Lim et al. 2012; Victor et al. 2021). Aggregated to the community level, if local arrests simply take low offending possession individuals off the streets for temporary periods of time, when they return to the community the local overdose rate could increase due to increased odds of overdose upon release from incarceration settings (Giftos and Tesema 2018). It is also possible that local arrests disrupt networks of social support among the existing market, networks that may otherwise protect users from dying of overdoses (Bouchard et al. 2018; Bennett et al. 2022; Byles et al. 2024; Clark et al. 2014; Cooper et al. 2005; Enteen et al. 2010; Mercer et al. 2021). People who use drugs or witness others using drugs can help each other to administer naloxone or can call emergency medical services in case of an overdose (Lankenau et al. 2013). Thus, the existing evidence examining the relationship between the criminal justice system and the opioid epidemic would suggest the alternative hypothesis:

- Collateral local consequences hypothesis 1b: More local arrests would contribute to more opioid overdoses locally (deaths and EMS).

### Law Enforcement and the Opioid Epidemic: Network Spillover Effects

Scholars interested in understanding how social phenomena are embedded in space have long had to reckon with the fact that neighborhoods are not isolated islands (Matthews and Yang 2013). Along with other social phenomena such as poverty or infant mortality, crime and crime-promoting processes tend to cluster together in geographically proximate communities (Hipp et al. 2012; Hipp and Williams 2020; Mears and Bhati 2006; Morenoff et al. 2001). Thus, when scholars investigate the success of place-based crime interventions, such as hot spot policing, they also consider the possibility of spillover effects (McDonald et al. 2024; Papachristos and Bastomski 2018; Tucker et al. 2012). On the one hand, increased police presence in one community could simply displace crime and drug use into neighboring communities, leading overall crime rates to remain the same (Ray et al. 2023; Wood et al. 2004). It is also possible, however, for the diffusion of crime-reducing benefits to occur (Weisburd and Telep 2013). Reviews of studies investigating spillover effects, broadly, find mixed results, as the displacement of crime or diffusion of crime-reducing benefits is context dependent, varying by the geographic unit of analysis, the type of the intervention or crime targeted, as well as a host of other macrosocial contexts (Telep et al. 2014).

While most research on spillovers has focused on *geographical* proximity effects, a growing body of literature is also calling attention to the potential for *social* spillovers (Graif et al. 2014; Levy et al. 2020; Newmyer et al. 2022). Just as communities are spatially connected by sharing a neighborhood boundary or border, they are also socially connected by the everyday flow of individuals across the city as they conduct their routine activities and commute for work. Scholars that have investigated crime have found that crime-promoting processes occurring in socially connected neighborhoods influence one another through social spillover effects (Graif et al. 2017; Levy et al. 2020). Just as a drug supply network may span across geographically proximate communities, these drug networks can also extend to more spatially distant neighborhoods that are socially connected by the everyday flow of individuals across space as they conduct their routine activities and commute for work.

When it comes to opioids, spillover processes could occur through the physical and social transmission of opioids and opioid misuse across space, as drugs and the individuals that use and sell them travel from one place to another. Arrests of drug suppliers in one community may disrupt drug supply chains that extend into neighboring communities. If a major supplier or distribution network is disrupted through arrests, it may temporarily reduce the availability of opioids locally and in nearby communities (Telep et al. 2014). This supply-side disruption could also contribute to reduced opioid misuse in surrounding or socially connected areas. Similarly, information, norms and attitudes about opioid misuse can move across communities along individuals’ friendships and mobility networks (Chaney and Rojas-Guyler 2015; De et al. 2007; Kwan et al. 2008; Mason et al. 2004). Individuals in neighboring communities may be socially connected to those in the focal community where arrests occur. Information about arrests and heightened law enforcement attention may spread through social networks, increasing awareness of the risks associated with opioid misuse. This social diffusion of awareness of police presence could influence behavior change in connected communities. The act of commuting and conducting one’s daily activities in neighboring communities could also mean that even if residents do not know about the increased risk of arrests from their social network, they may be made aware when conducting their routine activities (Kwan et al. 2008). Thus, the visibility of arrests in one community may increase the perceived risk of punishment among those who use or supply opioids not just locally but also in neighboring communities.

- Diffusion of benefits hypothesis 2a: Opioid arrests in the a) spatial and b) social network of a given community will contribute to fewer local opioid overdoses (deaths and EMS) in that community.

Alternatively, rather than benefits, risk may be diffusing along the spatial and social networks. Arrests in one community might contribute to the diffusion or displacement of opioids and the risk of opioid misuse to surrounding or socially connected communities. For example, law enforcement interventions in an area may prompt those who sell and use opioids to move some or all of their activities to spatially or socially proximate areas as individuals move to avoid the police. Indeed, more than just increasing fatal overdoses in the local area, Ray et al. (2023) finds that police drug seizures of illicit opioids in Marion County, Indiana increase the fatal overdose rate in geographically proximate areas. Thus, rather than reducing crime and overdoses, the misuse of opioids may simply be transferred into neighboring communities (Ray et al. 2023; Wood et al. 2004).

- Diffusion of risk hypothesis 2b: Opioid arrests in the a) spatial and b) social network of a given community will contribute to more local opioid overdoses (deaths and EMS) in that community.

We believe that countervailing forces are no doubt simultaneously at work, yet some forces may ultimately prevail. To the extent that these forces are weak or relatively equal to each other, the resulting pattern may be null.

### Heterogeneity in Network Spillovers on the Opioid Epidemic

Given the mixed results suggesting that both the displacement of crime and the diffusion of crime-reducing benefits can occur from criminal justice interventions (Telep et al. 2014), this study also investigates the potential heterogeneity in the spatial and social spillover of arrests on overdoses. We pay particular attention to variation based on 1) the fatality of the opioid overdose, 2) the severity of the opioid-related crime committed, and 3) the type of drug associated with the arrest. Prior research on the opioid overdose epidemic has placed a strong emphasis on fatal overdoses, with less attention on overdoses that do not result in mortality (Bohnert et al. 2011a, b; Erfanian et al. 2019; Friedman and Shover 2023; Hawk et al. 2015; Krawczyk et al. 2020). In this study we examine not just fatal overdoses, but also non-fatal opioid overdoses based on calls to Emergency Medical Services for an opioid overdose. We examine fatal and non-fatal opioid-related overdoses as two separate measures to allow for a broader picture of overdose incidents beyond those resulting in death. Second, it is likely that the spatial and social spillover mechanisms are different when the arrests are for the manufacturing and distribution of opioids, compared to when the arrests are for the possession of opioids. While there is some evidence that drug seizures (the supply side) lead to the displacement of opioid overdoses in Marion County, Indiana (Ray et al. 2023), it is unclear if supply-side arrests lead to the displacement of overdoses or the diffusion of overdose-reducing benefits or whether arrests for possession result in spillovers. Finally, the use of synthetic opioids, such as fentanyl, is associated with a higher risk of overdose and death than the use of heroin (CDC 2024; Friedman and Shover 2023; Hopwood et al. 2020; Pichini et al. 2018). Moreover, the mortality rate associated with heroin has remained at a relatively steady, non-increasing rate, while the mortality rate associated with synthetic opioids has increased dramatically over the last decade (CDC 2024). Thus, it is possible that the relationship between arrests and the fatality of the overdose may also vary with the type of drug offense being committed.

## Methods

### Data

To investigate the relationship between criminal justice drug intervention practices and the opioid epidemic, we combine multiple sources of data on the city of Chicago. Opioid-related health data was obtained from the Chicago Health Atlas, which provides data on the total number of individuals who died from an opioid-related drug overdose in each community area as reported by the Cook County Medical Examiner (CCME) between 2016 and 2019. Chicago Health Atlas also provides the number of EMS responses to opioid-related overdoses by Chicago Fire Department ambulances each year from 2016 to 2019. This data is reported from the Chicago Fire Department, National Emergency Medical Services Information System (NEMSIS). Arrest reports are provided by the Chicago Police Department’s CLEAR (Citizen Law Enforcement Analysis and Reporting) system^5^ during the entire period of study. We use data from the 2010 to 2014 5-year estimate American Community Survey (ACS) to obtain total population counts for each community area for the creation of rates.^6^ Additionally, the City of Chicago Homeless Point-in-Time (PIT) Count and Survey Report provides annual statistics on homelessness across the city’s community areas (City of Chicago 2024).^7^ While the period 2016 to 2019 was selected partially due to data availability constraints, the period aligns with the spike in overdoses that contributed to the declaration of the epidemic as a national public health emergency (CDC 2019).

To investigate potential social spillovers we examine population mobility based on commuting statistics, consistent with other research (Graif et al. 2017; Newmyer et al. 2022). The Longitudinal Household Employer Dynamics (LEHD) Origin–Destination Employment Statistics (LODES) is a data collection effort sponsored by the U.S. Census to provide information on the geographic location of employers and their employees (Graham et al. 2014). The Census provides annual commuting flow statistics between work and residential communities, which we conceptualize as social connections between communities. We take Chicago’s community areas as our unit of analysis due to data availability, but also consistent with prior research that has shown effective interventions at higher levels of geography than hot spots (Mazerolle et al. 2020).

### Measures

#### Dependent Variables

Our analysis on the opioid epidemic investigates two dependent variables. Consistent with other research on the opioid epidemic, we first investigate fatal opioid overdoses with the total number of individuals reported by the Cook County medical examiner to have died from an opioid-related drug overdose each year. This measure is available for each community area and represents the total number of individuals that died in the location, even if they did not reside in Chicago. With this measure we create a fatal opioid overdose rate per 100,000 for each of Chicago’s 77 community areas between 2016 and 2019. Our second measure is more expansive, capturing the full presence of the opioid epidemic in Chicago with the total number of times the Chicago Fire Department ambulances responded to opioid-related overdoses each year. From this data we create an EMS-response opioid-related overdose rate per 100,000 for each community area between 2016 and 2019.

#### Independent Variables

Law enforcement activities related to opioid activities are operationalized as opioid arrests for possession and manufacturing or distribution, as reported by the Chicago Police Department. The analysis first investigates whether arrests in a focal community prevents opioid overdoses occurring in that same community. We investigate four different types of arrests: 1) arrest for possession of heroin, 2) arrest for possession of synthetic narcotics, 3) arrest for manufacturing or distribution of heroin, and 4) arrest for manufacturing or distribution of synthetic narcotics. The Uniform Crime Reporting (UCR) program defines heroin as white powder, tan or brown tar, and black tar. The UCR defines synthetic narcotics as manufactured narcotics that can cause true addiction such as fentanyl. If, for example, heroin is laced with fentanyl it will be defined as an arrest for synthetic narcotics (FBI 2019). For each of the four arrest measures we create an arrest rate per 100,000.

The analysis also investigates the spatial spillover of policing practices, assessing whether arrests in spatially proximate communities prevent or displace opioid overdoses in the local community. To investigate the role of spatial spillovers we create spatially lagged measures of arrest rates. We create these measures by first defining a row-standardized spatial weights matrix using the queen criterion. The queen spatial weights matrix is a contiguity-based matrix that considers every spatial unit that shares a portion of the border, even just a single point or vertex, as a spatial neighbor. After determining which communities are spatial neighbors, we use the spatial weights matrix to calculate the spatial lag, which represents the average arrest rate in the neighboring communities. The spatial lag can be expressed as Wx, where W represents the spatial weights matrix defining which communities are neighbors, and x represents the arrest measure. We create a spatial lag for each arrest measure.

The primary focus of our analysis is on whether social spillovers occur, with arrests either preventing or displacing opioid overdoses in socially connected communities. We investigate social spillovers by defining a row-standardized weights matrix which represents social neighbors, rather than spatial neighbors. Consistent with other work investigating social spillovers and patterns of population health (Newmyer et al. 2022), we create a social weights matrix using commuting flows. Using the LODES data from 2016, we create a 77 by 77 valued matrix representing the percentage of residents which commute from the home community to the work community. We convert the asymmetric valued matrix into a symmetric, dichotomous matrix representing social neighbors by defining work communities which receive at least 0.5% of the home communities’ residents as the home communities’ social neighbors. Thus, if at least 0.5% of Englewood’s residential population is commuting to Hyde Park for work, then we would consider Hyde Park and Englewood to be socially connected. We chose to use a 0.5% population commuting threshold to remain consistent with other research on commuting flows between community areas in Chicago finding 0.5% represents a meaningful population cutoff (Graif et al. 2019; Newmyer et al. 2022; Evans et al. 2023). This value represents, on average, 175 daily commuters between communities. When using smaller thresholds, most of the communities become connected together, while when using larger thresholds most communities are only connected to a few work hubs such as O’Hare or the Loop. With the 0.5% threshold, communities are, on average, connected to 7 other communities. Using the social neighbors weights matrix, we create a socially lagged measure of arrests, representing the average arrest rate in the socially connected communities. We create a social lag for each arrest measure.

#### Controls

Our analysis accounts for two time-varying community characteristics which may confound the relationship between law enforcement responses to substance use and the opioid epidemic: crime and homelessness. Research has shown that prior crime involvement can contribute to future crime and drug use both at the individual level (Lim et al. 2012) and at the neighborhood level (Bohnert et al. 2011b). Homelessness is also a risk factor for drug use and opioid overdose (Enteen et al. 2010). For each community area we total all crime reports, independent of whether an arrest took place, from the Chicago Police Department to create a crime rate per 100,000 for each year under observation. With the Point-in-Time estimates we create a community area homelessness rate per 100,000 for each year. We also account for time as a continuous measure.

### Analytic Strategy

We apply fixed effects spatial autoregressive models. Spatial models account for the tendency for social phenomena in spatial units that are more proximate to one another to be correlated, i.e., spatially dependent. The global Moran’s I test is used to assess the appropriateness of applying a spatial autoregressive model, testing for the presence of spatial dependence among a variable. The global Moran’s I coefficient like a traditional correlation coefficient, has values ranging from −1 to +1. A statistically significant positive value indicates that spatial patterns in the observed data exhibit more clustering than would be expected under random distribution, suggesting the presence of meaningful spatial dependence (Anselin 2003). Both dependent variables, the fatal opioid-related overdose rate and the EMS-response opioid-related overdose rate, are spatially dependent in each year of observation.^8^ We employ spatial lag models as they model the spatial dependence in the dependent variables as a spatial spillover process. Similar to the approach discussed above to model the spatial spillover of arrests, the spatial lag model accounts for the spatial spillover of opioid overdoses from the neighboring communities into the focal community. Postestimation tests indicate that incorporating a spatial lag of the dependent variables is the most appropriate modeling strategy (Anselin et al. 1996; Elhorst 2014).^9^ The spatial lag models use a row-standardized queen spatial weights matrix.

As our data is longitudinal, we use fixed effects models to account for the stable heterogeneity that exists over time between units. The longitudinal structure of the data helps account for the temporal causal ordering. The fixed effects approach reduces the likelihood of omitted variable bias by accounting for time-constant unobserved differences between communities (Allison 2009).^10^ We use the *spxtregress* command in Stata to estimate all models (StataCorp 2019). The following equation represents the Lee and Yu (2010) SAR model for panel data with fixed effects:

$$y_{nt}=\lambda Wy_{nt}+X_{nt}\beta +c_{n}+u_{nt}\;u_{nt}=\rho Mu_{nt}+\nu_{nt}t=1,2,\ldots ,T$$

where y represents the opioid overdose rate in community area n for year t. The 77 × 77 spatial weights matrix is represented by W and M, so $Wy_{nt}$ represents the spatial lag of opioid overdose rates resulting from the spatial weights matrix multiplied with y and $Mu_{nt}$ represents the spatial lag of disturbances resulting from the spatial weights matrix multiplied with disturbances.^11^ The time-varying covariates are represented by X, which includes local arrest rate and the spatial and social lag of arrests as well as the other control variables; lambda is a scalar; β is a vector of regression coefficients; and $c_{n}$ represents the vector of community fixed effects.^12^ Finally, u represents the spatially lagged error, and v represents a vector of independent and identically distributed disturbances.

## Results

### Descriptive Statistics

Table 1 presents the descriptive statistics of opioid-related overdoses and arrests in Chicago, averaged between 2016 and 2019. There is a stark difference in magnitude between overdose incidents and fatal overdoses. Across the 77 community areas, the average EMS-response opioid-related overdose rate was 10 times higher than the fatal opioid-related overdose rate, at 329 individuals per 100,000 compared to 31 individuals per 100,000. The average arrest rates for heroin are higher than the arrest rates for synthetic narcotics, as are arrests for possession in comparison to manufacturing and distribution. The stark difference in overdose rates by fatality and the variation in the average arrest rates by the severity of the arrest and type of drug associated with the arrest support our intuition to examine potential heterogeneity in the relationship between arrests and opioid overdoses.

**Fig. 1:**
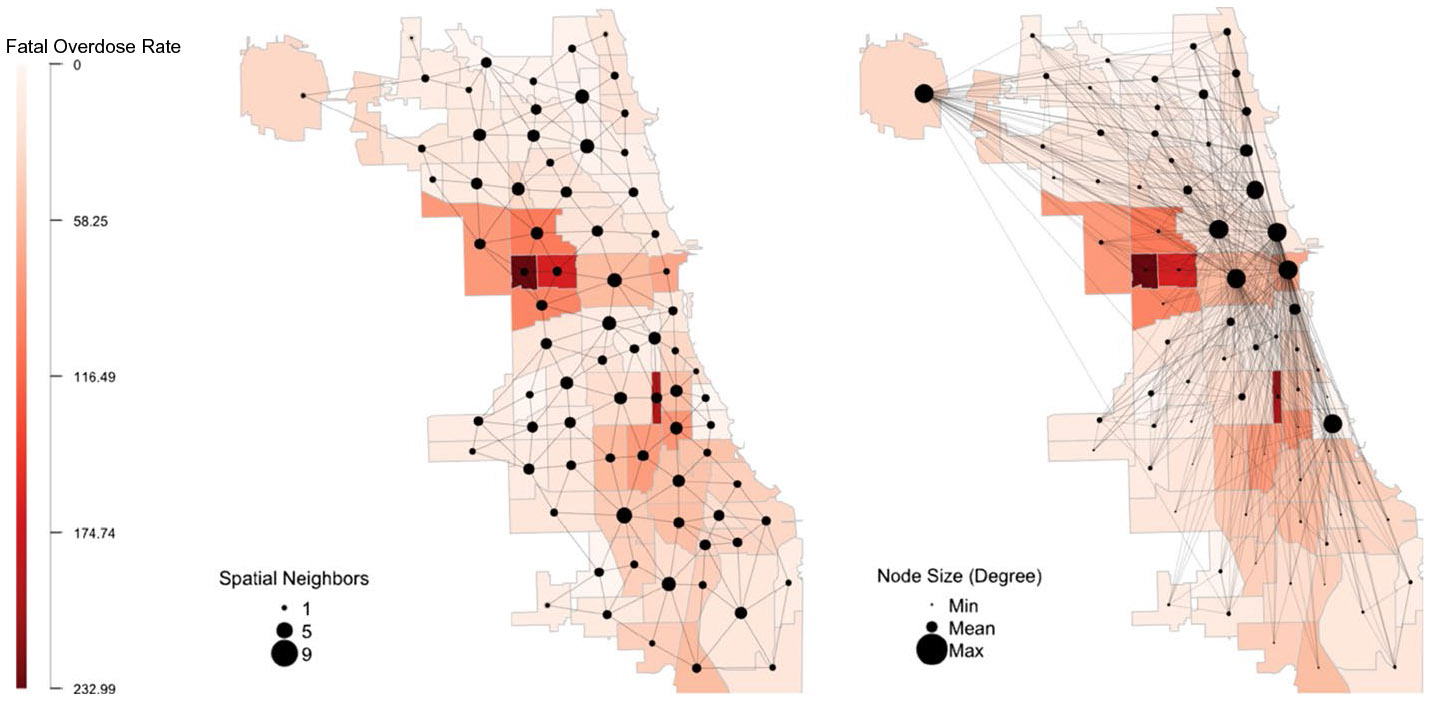
Map of Fatal Opioid-Related Overdose Rates in 2019 across Chicago’s 77 Community Areas with Community Ties by a) Spatial Proximity and b) Social Proximity. Note: Rates are per 100,000. Communities are colored on a continuous red scale where darker shades represent higher fatal opioid-related overdose rates. Spatial proximity is defined using the Queen 1 criteria where every community sharing a contiguous border is defined as spatial neighbors with the opportunity to share spatial spillovers. Social proximity is defined using commuting flows where a community is a social neighbor with the opportunity to share social spillovers if they share at least 0.5% of their residential population commuting to or from work. Nodes are sized by the number of a) spatial neighbors or b) social neighbors

Figure 1 presents a map of fatal opioid-related overdose rates in 2019 across Chicago’s 77 community areas visually demonstrating which communities are connected via a) spatial proximity and b) social proximity. The communities are shaded on a continuous red scale where darker shades represent communities with higher opioid death rates. Each grey edge connects a community if they are a) spatial neighbors or b) social neighbors based on commuting ties. The nodes are sized by the number of either a) spatial or b) social neighbors where smaller nodes represent fewer connections, and larger nodes represent more connections. Figure 1 demonstrates how commuting ties connect communities that are both spatially near and far, providing opportunities for the social spillover of both crime and criminogenic-promoting or preventative forces. Figure 1 also demonstrates that communities with high overdose rates tend to spatially cluster together in close geographic proximity.

### Arrests for Possession and Opioid Overdoses

Tables 2 through 5 present the results from the fixed effects spatial autoregressive models estimating opioid overdose rates in Chicago between 2016 and 2019. The first two tables assess the relationship between arrests for possession and opioid overdoses while the second two tables assess the relationship between arrests for manufacturing/distribution and opioid overdoses. Within each table, the first set of models estimate the EMS-response opioid overdose rate, and the second set of models estimate the fatal opioid overdose rate. Model 1 includes the local arrest rate and the average arrest rate of the spatially proximate communities to assess whether there is a spatial spillover of arrests on opioid overdoses. Model 2 includes the local arrest rate and the average arrest rate of the socially connected communities to assess whether there is a social spillover of arrests on opioid overdoses. Finally, model 3 includes the local arrest rate, the spatial spillover of arrests, and the social spillover of arrests together to assess the full relationship between arrests and overdoses. All models include time-varying controls and the spatial lag of opioid overdoses. All measures presented in the models are standardized to a mean of 0 and a standard deviation of 1.

Table 2 presents the results assessing the relationship between arrests for the possession of heroin and opioid overdoses. In the first panel analyzing the EMS-response opioid overdose rate, model 1 finds that a one standard deviation increase in the local arrest rate for heroin possession increases the EMS-response opioid overdose rate by 0.360 standard deviations (p < 0.001), and a one standard deviation increase in arrests for heroin possession in spatially connected communities increases the EMS-response opioid overdose rate by 1.620 standard deviations (p < 0.001). Arrests for heroin possession serve to increase opioid overdoses rather than decrease them in local and neighboring communities. In model 2, we continue to find a significant relationship between the local arrest rate for heroin possession and EMS-response opioid overdoses. We also find that increasing the arrests for heroin possession in socially connected communities increases the local community’s overdose rate. However, when all three measures are included in model 3, the social displacement of overdoses disappears. Rather, there is a strong spatial spillover effect, with arrests displacing opioid overdoses into neighboring communities. Though commuting flows connect more physically distant communities, it is also often the case that communities which are spatially proximate are socially connected by the everyday flow of commuters. Thus, model 3 suggests that the positive relationship found in model 2 for the social spillover of arrests was reflecting the spatial spillover of arrests on overdoses.

We also find across all three models that a one standard deviation increase in the total crime rate increases the EMS-response opioid overdose rate by a standard deviation. Overall, the results from the first panel of Table 2 suggest that arrest for the possession of heroin increases the total opioid overdose rate in the local community as well as in spatially proximate communities, displacing opioid misuse and overdoses.

In contrast to the findings for the total opioid overdose rate, represented by the EMS-responses for opioid overdoses, we find a null local and spatial spillover relationship between arrests for the possession of heroin and fatal opioid overdoses. Rather, we find a strong social spillover of arrests on overdoses, where arrests for the possession of heroin displaces opioid misuse and overdoses into socially connected communities. In the first model there is a nonsignificant negative relationship between arrests in the local community and fatal overdoses, and a significant positive social spillover relationship of arrests on fatal overdoses, suggesting that increasing arrests in the local community displaces fatal overdoses into geographically proximate communities. However, the spatial spillover relationship disappears when we account for the social spillover in model 3. The final model indicates that increasing arrests for the possession of heroin in socially connected communities serves to displace opioid misuse and fatal overdoses into socially proximate communities.

We also find a significant spatial lag of fatal opioid overdoses, suggesting that the fatal overdose rate in spatially proximate communities serves to increase the fatal overdose rate in the local community. There is also a positive relationship between the homelessness rate and crime rate with the fatal opioid overdose rate, consistent with the existing literature (Enteen et al. 2010; Lim et al. 2012). Overall, the results suggest that arrests for the possession of heroin in the local community and in spatially proximate communities have the unintended consequence of increasing the *total* opioid overdose rate in the local community, and arrests in socially proximate communities serve to increase the *fatal* opioid overdose rate in the local community.

Table 3 presents the results assessing the relationship between arrests for the possession of synthetic narcotics and opioid overdoses. Similar to the findings presented in Table 2 for the possession of heroin, there is a significant local and spatial spillover of arrests for the possession of synthetic narcotics on the EMS-response opioid overdose rate. Model 1 suggests that a one standard deviation increase in the local arrest rate and the average arrest rate of spatially connected neighbors for possession of synthetic narcotics increases the EMS-response opioid overdose rate in the local community by 0.129 (p < 0.001) and 0.182 (p < 0.01) standard deviations, respectively. There is no social spillover effect of arrests for the possession of synthetic narcotics on the opioid overdose rate. The first model in the second panel similarly finds that increasing the local arrest rate for the possession of synthetic narcotics increases the fatal opioid overdose rate by 0.133 standard deviations (p < 0.001). In contrast with the total opioid overdose rate, there is no spatial or social spillover of arrests for possession of synthetic narcotics on fatal overdoses.

Together, the results suggest that arrests for opioid misuse reflected by the possession of heroin or synthetic narcotics serve to increase the *total* overdose rate in a community. Not only do overdoses increase from local arrests, but also from arrests in spatially proximate communities, perhaps diffusing or displacing opioid misuse. Local arrests for the possession of synthetic narcotics also increase the *fatal* overdose rate in the local community, and arrests for the possession of heroin in socially connected communities increase the fatal overdose rate. The spatial and the social spillovers of risk from arrests for synthetic narcotics possession do not have a significant association with local levels of fatal overdoses.

### Arrests for Manufacturing/Distribution and Opioid Overdoses

Table 4 presents the results assessing the relationship between arrests for the manufacturing/distribution of heroin and opioid overdoses. Consistent with the results for possession discussed above, we find a significant positive relationship between arrests for the manufacturing/distribution of heroin and the EMS-response opioid overdose rate. Arrests in the local community as well as in spatially proximate communities increase the local overdose rate, with null effects on the fatal opioid overdose rate. In the first panel, model 3 indicates there is also a negative relationship between arrests for manufacturing/distribution of heroin in socially connected communities and overdoses. In contrast to the findings for the local and spatial spillover of arrests, increasing the arrests for the manufacturing/distribution of heroin in socially connected communities helps to prevent opioid overdoses in the local community, potentially disrupting the drug supply network from more spatially distant though socially connected communities. Thus, while the local arrests for the manufacturing/distribution of heroin increases local overdoses and overdoses in spatially proximate communities, it has the potential to disrupt the drug supply networks in socially connected communities and lower opioid overdose rates.

Table 5 presents the results assessing the relationship between arrests for the manufacturing/distribution of synthetic narcotics and opioid overdoses. In contrast to findings for heroin and possession, increasing local arrests for the manufacturing/distribution of synthetic narcotics decreases the local EMS-response opioid overdose rate. There are no spatial or social spillovers of arrests for the manufacturing/distribution of synthetic narcotics on the EMS-response opioid overdose rate. However, models 2 and 3 for fatal overdoses find that increasing the arrests for manufacturing/distribution of synthetic narcotics in socially connected communities serves to increase the local fatal opioid overdose rate, similar to the finding in Table 2 for arrests for the possession of heroin. These results suggest that opioid misuse and fatal overdoses are displaced into socially connected communities after arrests for the manufacturing/distribution of synthetic narcotics increases. While there is no spatial spillover, opioid misuse is displaced into more spatially distant but socially connected communities. These results contrast with those for the manufacturing/distribution of heroin. While disrupting the drug supply network of heroin has crime-reducing benefits and reduces fatal overdoses in socially connected communities, disrupting the drug supply network of synthetic narcotics simply displaces the drug supply network and associated fatal overdoses.

Together, these results suggest that arrests for possession of heroin increase the EMS-response opioid overdose in local communities and in spatially proximate communities. While there is no social spillover of arrests for possession on EMS-response overdoses, there is a social spillover of arrests for heroin possession on fatal overdoses. There is also a social spillover of arrests for manufacturing/distribution on EMS-response overdoses and fatal overdoses. Arrests for the manufacturing/distribution of heroin has a social spillover of crime-reducing benefits, decreasing the EMS-response overdose rate in socially connected communities. However, the arrests for the manufacturing/distribution of synthetic narcotics and the possession of heroin displaces opioid misuse and fatal overdoses into socially connected communities. These results suggest a complex spatial and social spillover relationship between arrests and overdoses, dependent on the severity of arrest, drug associated with the arrest, and fatality of the overdose.

### Supplementary Results

Though not included in the main set of analyses, we also explored how our definition of a commuting tie cutoff of 0.5% of residents moving daily between a residential and work community influences the social spillover of arrests on overdoses. In Appendix Table A5 we replicate the results from Tables 2 through 5 using commuting tie cutoffs of 0.25% and 2%. The results suggest that using a weaker tie definition of 0.25% of residents provides similar results on the social spillover of arrests on overdoses as presented in the main results. Using a stronger tie definition, however, does not result in a social spillover of arrests on overdoses. These results are consistent with expectations as using a stronger threshold to define a commuting tie means many communities are only connected to the two large work hubs in the city, O’Hare and the Loop. These supplemental analyses suggests that the strength of social connections between communities, defined as the percentage of residents commuting to and from work is an important determinant of whether processes of social spillovers can occur between connected communities.

We also explored whether the relationship between arrests and overdoses changes in 2020 during Covid-19 with an additional year of data. We do not include 2020 in our main analysis because population mobility looked fundamentally different during the Covid-19 pandemic when stay-at-home orders required only fundamental workers leave their residence to commute for work. Moreover, stay-at-home orders meant limited daily activities of individuals, more broadly, meaning that fewer residents were on the streets and social networks and interactions were more limited. Despite stay-at-home orders, the opioid crisis spiked even higher during the pandemic. These changes lead us to suspect a fundamentally different relationship between arrests and opioid overdoses in the year 2020. Appendix Table A6 presents the results replicating Tables 2 through 5 using a cross-sectional spatial lag model for the year 2020. Similar to the findings for the years prior to the Covid-19 pandemic, in 2020 there continues to be a strong and positive local and spatial spillover relationship of arrests on the total overdose rate. In contrast to the prior years, there is also a strong local and spatial spillover relationship of arrests on fatal overdoses. We also find a social spillover of arrests for synthetic narcotics possession, but the social spillovers are less salient than in the prior years, which is consistent with expectations since there were fewer individuals commuting to and from work.

Finally, we also explore how total opioid-related arrests, summarized as the total arrests for both heroin and synthetic narcotics influenced opioid-related overdose rates in the local, spatially proximate, and socially connected communities. Similarly, we investigate total opioid-related arrests by summarizing the total arrests for both possession and manufacturing/distribution. Appendix Table A7 compares patterns of arrests for all opioids in comparison to patterns specifically for heroin and synthetic narcotics, in addition to examining the relationship between all drug-related arrests and opioid-related overdoses. The patterns for the overall opioid-related arrests are most similar to that for heroin arrests, where increasing the local opioid-related arrest rate contributed to an increase in the local and spatially proximate EMS-response opioid-related overdose rate. Opioid-related arrests also increased the fatal overdose rate in socially connected communities. Results for all drug-related arrests are similar to that for all opioid-related arrests, suggesting that law enforcement strategies to curb drug use may have the opposite unintended effect of increasing overdoses and health-related harm.

## Discussion

Our findings suggest that law enforcement interventions, represented through opioid-related arrests, can have the unintended consequence of increasing opioid-related overdose rates, including fatal overdoses. We find that law enforcement interventions have the potential to backfire not only on local communities, but also on their spatially and socially proximate networks. In the following, we first discuss the local and spatially proximate relationships between arrests and overdoses, followed by a discussion of spillover relationships between communities embedded within socially proximate networks based on routine population mobility.

### Local and Spatially Proximate Relationships

The dominant pattern found across the analyses was that criminal arrests for opioid-related possession and arrests associated with heroin contributed to increases in EMS-response opioid-related overdoses in a local community and its spatially proximate neighbors. Increases in the local arrest rate for the possession of heroin and synthetic narcotics and for the manufacturing/distribution of heroin were associated with increases in the local EMS-response opioid-related overdose rate. Similarly, arrests for the possession of synthetic narcotics contributed to increases in the fatal overdose rate in the local community. These observed patterns are consistent with expectations of unintended health risks from contact with the police and criminal justice system (Krawczyk et al. 2020; Lim et al. 2012; Victor et al. 2021; Zhang et al. 2022). It is possible that police crackdown activities prevented opioid users and their friends and kin from seeking help or healthcare support for fear of being arrested themselves, as suggested by prior research (Cooper et al. 2012; Ostrach et al. 2022). Arrests and associated police crackdown strategies have been shown to disrupt existing and potential networks of support among users, which also prevents users from accessing harm reduction strategies that might otherwise prevent overdoses (Bennett et al. 2022; Bouchard et al. 2018; Byles et al. 2024; Cooper et al. 2005; Mercer et al. 2021).

It is notable that there was a stronger and more consistent relationship of overall opioid-related arrests with the total opioid overdose rate represented by EMS responses, compared to fatal overdoses (see Appendix Table A7). This pattern suggests that while criminal arrests for opioid misuse may increase the incidence of opioid overdoses requiring EMS response, it may not substantively increase overdose fatalities. These findings may reflect that EMS responders, equipped with opioid antagonists like Narcan, are often able to successfully reverse the fatal consequences of overdoses (Chicago Department of Public Health 2021). Moreover, the fact that more opioid arrests increase rather than decrease EMS-response overdose rates, raises important questions about the public health implications of relying on criminal justice approaches to the opioid epidemic. Non-criminal justice prevention strategies along with harm reduction strategies are likely more beneficial.

While not as prominent a pattern across the analyses, the results also suggest that arrests for the manufacturing/distribution of synthetic narcotics contributed to lower levels of EMS-response opioid overdoses in the local community. This is consistent with initial expectations that criminal justice activities would lead to crime prevention and decreased drug use and overuse (Braga et al. 2017; Chalfin and McCrary 2017; Nagin 2013). It is possible that police crackdown on synthetic narcotics’ manufacturing and distribution led to the disruption of the drug supply chain and distribution network (Eggins et al. 2020; Holland et al. 2023; Mazerolle et al. 2020) or functioned as related forms of social control, discouraging current and potential users from acquiring and misusing opioids (Braga et al. 2017; Sampson 1986). It is notable that arrests for synthetic narcotics are the only ones that consistently functioned as intended at a local level. It is possible that, because they are less frequent, they contribute less to a sense of harassment in the general population and are less likely to significantly disrupt networks of support among users.

It is also informative that *spatial* spillovers contributed to increases in a community’s total opioid overdose rate, represented by EMS responses to overdoses. This pattern was consistent across arrests for possession and for the manufacturing/distribution of heroin. With respect to fatal overdoses, we found detrimental effects from spatial spillovers from arrests for the possession of opioids and all drug-related arrests (see Appendix Table A7). Overall, the patterns of spatial spillovers were observed to track closer to the patterns for local arrests compared to the patterns for socially connected networks, which is more complex and discussed below. The coefficients estimating EMS overdoses were larger in magnitude for spatial spillovers than local effects in the case of heroin possession arrests and more similar in size for synthetic possession and heroin manufacturing/distribution.

### Ecological Network Spillovers

Overall, the results suggest that arrests for the possession or manufacturing/distribution of opioids can have spillover effects on socially connected communities. These findings are consistent with the rapidly growing thread of research showing that communities are not independent islands as assumed for many decades in the neighborhood effects space (Mears and Bhati 2006; Graif et al. 2014). They highlight the significance of place-to-place connections through population mobility ties in transmitting other forms of health and safety risks, such Covid-19 infection risk, STIs, and domestic and sexual violence (Graif et al. 2021; Kelling et al. 2021; Newmyer et al. 2022; Seto et al. 2022). The results suggest that, just as drug suppliers and consumers or information about drug access can travel between *geographically* proximate communities (Telep et al. 2014; Wood et al. 2004), daily commuting flows also can provide opportunities for drug access and information about drug misuse to travel between *socially connected* communities, including those that are geographically distant from each other. Residents who commute between communities may serve as bridges, perhaps transmitting drugs but also attitudes and behaviors protecting against drug misuse, or information about law enforcement activities.

The current study advances the literature through a focus on commuting ties. Commuting for work is predominantly prosocial by nature, and thus likely provides a conservative test of the association between drug arrests and overdoses. Still, commuting flows map onto different forms of transportation and communication networks between communities, which facilitate the movement and interactions of people for work as well as non-work reasons, including the movement of drug suppliers and users. Indeed, research has shown that people who sell drugs at the street-level often do so concurrently with legitimate employment (Reuter and MacCoun 1992; Nguyen et al. 2023). The boundaries between prosocial and antisocial networks can sometimes blur, such as when doctors illegally sell prescription opioids or service workers leverage customer relationships to expand their network of drug clientele (Gershowitz 2020; Novick 2019). While more research is needed on the underlying mechanisms of risk transmission, such examples can enhance our understanding of how commuting ties can facilitate networks relevant for encouraging or discouraging drug use between communities that are geographically distant though socially proximate.

Across all models, the findings highlight important heterogeneity in network spillover effects of arrests on opioid-related overdoses in socially connected communities, showing that social network proximity defined based on commuting mobility enabled at times the diffusion of opioid overdose risk while, at other times, contributed to prevention. First, arrests for the possession of opioids and heroin, in particular, contributed to increases in fatal overdoses in socially connected communities. Like the unintended consequences of arrests on the EMS-response overdose rate in local and spatially proximate communities, this finding is consistent with expectations of crime and health risk diffusion across intercommunity networks in space (Graif et al. 2014; Kelling et al. 2021; Levy et al. 2020; Newmyer et al. 2022; Seto et al. 2022). Similarly, arrests for the manufacturing/distribution of synthetic narcotics had the unintended consequence of increasing fatal overdose risk in socially connected communities. In light of the beneficial finding of decreasing the EMS overdose risk from this type of arrest in local communities, the social spillover perhaps indicates a phenomenon of displacing fatal opioid overdose risk away from local communities into socially connected communities (Wood et al. 2004). This is consistent with the literature on crime displacement which has highlighted concerns that interventions to stop crime at specific hotspots may displace the crime risk to other places (Telep et al. 2014).

We note that the social spillover relationship that emerged between opioid arrests and fatal overdoses contrasts with the null local and spatial spillover relationships. It may be that disrupting the supply and distribution chain across the network leads local users to look for alternatives in less familiar, more distant places, perhaps relying instead on even less safe drug sources. The use of less familiar networks may result in fatal overdoses due to fentanyl contamination of recreational drugs that is more likely when users need to tap into alternative, less trusted drug supply networks (Cristiano 2022; Hopwood et al. 2020; Pichini et al. 2018).

Importantly, the findings also showed that arrests in socially connected communities for the manufacturing/distribution of opioids and heroin, in particular, successfully prevented local opioid overdoses, as measured by EMS responses. This pattern is consistent with expectations for the diffusion of health benefits across networks of population mobility, where law enforcement activities that disrupt the network of opioid supply and distribution can have ripple effects on socially connected communities (Eggins et al. 2020; Holland et al. 2023; Mazerolle et al. 2020; Telep et al. 2014). As opioid drug suppliers are not widely distributed across all communities in the city, these findings indicate that cracking down on specific manufacturing/distribution hot spots may cut off the flow of opioids to more distant but connected communities. However, this finding does contrast with findings of detrimental social spillovers for fatal overdoses, which suggests complex social spillovers dependent on the type of opioid being manufactured/distributed as well as the fatality of the overdose.

## Limitations and Directions for Future Research

The study has several limitations which offer important areas for future research. First, it only examines community areas in Chicago. Future studies will benefit from reiterating the analyses with spatial units of different sizes and in cities with different histories related to the opioid crisis. It would also be valuable for future research to focus on rural communities as their specific needs and spillover patterns are likely to differ from urban patterns (Lister et al. 2020). Second, this study only includes commuting mobility ties to understand connections between communities. Future research should consider other types of routine mobility that could serve as ecological bridges between communities to offer a wider view of activities and settings of possible interpersonal interactions and influence across communities. For example, routine travel to certain bars can increase access to drugs, while traveling to certain churches or health organizations can deter people from using drugs. Third, our analysis specifies commuting flows as a symmetric, dichotomous network. Future research should investigate whether the directionality of population flows differentially influences social spillover processes. Investigating asymmetrical ties would uncover whether there is variation in the relationship between arrests and overdoses based on whether spillover processes occur by bringing work-based exposures home, or if residents bring home-based exposure to their work community. We also urge scholars to further explore the strength of ties and whether critical thresholds in the volume of population flows exist for the diffusion of drug use and overdoses.

Fourth, the study is not based on a randomized experimental design, limiting a strict causal interpretation of the findings. However, it is important to note that the inclusion of covariates helped control for possible time-varying confounders, and using a longitudinal panel design and fixed effects modeling helped to establish and control away time-invariant factors. Fifth, while EMS data are likely the best proxy for nonfatal overdoses, we note that given the increasingly widespread availability of opioid antagonists like Narcan, nonfatal overdoses that are managed by individuals or peers without contacting emergency services would not be captured in these data. In the context of illicit markets, those fearing arrests may be reluctant to call 911.^13^ Sixth, due to data limitations, this study utilized residential population counts as the denominator for rates of overdoses and arrests. Arrests and overdoses within a community can involve non-residents, which is an important consideration in spatial research (Johnson et al. 2020). Though our sample is subject to the unknown denominator problem (Morrison et al. 2020), this study advances prior work by accounting for non-residential exposures due to population mobility between different areas by including commuting flows. Finally, this study focused on opioid overdoses as explained by opioid-related arrests. Future studies will benefit from examining more closely the impacts of *overall* drug arrests on opioid and other drug overdoses. Preliminary results presented in Table A7 suggest a significant role for social network spillover, but digging deeper will be important for future research.

## Conclusion

This study found that opioid arrests led, more often than not, and especially in the case of arrest for opioid possession, to increased risk of opioid overdoses. These deleterious effects were observed within a community as well as across other communities that were spatially proximate or connected through routine population mobility networks. These findings add to a growing number of studies finding unintended local effects of law enforcement activities and collateral consequences of criminal justice contacts (Krawczyk et al. 2020; Lim et al. 2012; Victor et al. 2021; Zhang et al. 2022). The findings advance existing knowledge by also highlighting that the deleterious impact on health apply beyond individuals (Kopak et al. 2018; Krawczyk et al. 2020; Lim et al. 2012; Victor et al. 2021), to affect the broader community (Bohnert et al. 2011b). Importantly, we show for the first time, to our knowledge, that the effects of opioid arrests can spillover beyond geographic proximity (Bohnert et al. 2011b; Ray et al. 2023; Telep et al. 2014; Wood et al. 2004) to affect overdose risk in connected communities across the entire city.

The findings underscore the complexity of the opioid epidemic and the limitations of relying on criminal justice approaches to drug misuse. While some silver linings emerged from these findings, as for certain opioid arrests the results matched the intended beneficial effects of lowering opioid-related overdoses, more often it was the case that arrests significantly increased the risk of opioid-related overdoses. This suggests that to effectively combat the opioid epidemic, a comprehensive strategy is needed that combines law enforcement efforts more targeted on the manufacturing/distribution of opioids with evidence-based public health interventions targeted at harm reduction, such as expanding access to medication-assisted treatment. Indeed, the Chicago Department of Public Health launched programs like the public health vending machine that distributes harm reduction tools and hygiene kits to communities with high overdose rates, as well as setting up a helpline for those seeking treatment for opioid use disorder (OUD). The evaluation of such programs might benefit from a spatial and social network approach.

Overall, the findings have important implications for future research and policy by highlighting that intercommunity networks across the city facilitate the transmission of opioid overdose risk and protections, a phenomenon that remained otherwise hidden when using standard neighborhood models or spatial models. The results suggest that public health officials and policymakers must consider the spatial networks of mobility when addressing the opioid epidemic. Continuing to improve our understanding of the spatial and population mobility network dynamics of opioid misuse as well as the potential spillover effects of interventions will enable policymakers to develop more effective and coordinated responses to the ongoing opioid crisis. As studies begin to use spatial networks approaches to build a better understanding of the ripple effects of law enforcement activities or harm reduction programs on opioid overdoses across population mobility networks, it will ultimately benefit not just the directly affected communities but the entire city at large.

## Supplementary Material

supplement

**Supplementary Information** The online version contains supplementary material available at https://doi.org/10.1007/s10940-025-09612-y.

## Figures and Tables

**Table 1:** Descriptive statistics of overdoses and arrests in Chicago, 2016–2019

	Mean/Prop.	Min.	Max.	*n*
Opioid Overdose Rates				
EMS-Response Overdose	329.72	.00	5792.96	308
Fatal Overdose	31.19	.00	243.58	308
Arrest Rates				
Heroin Possession	107.50	.00	2441.09	308
Heroin Manufacture/Distribution	51.20	.00	2043.95	308
Synthetic Narcotic Possession	14.55	.00	190.63	308
Synthetic Manufacture/Distribution	2.49	.00	180.04	308
Time-Varying Controls				
Crime Rate	10682.33	2310.56	36610.30	308
Homeless Rate	41.17	.00	1145.44	308

Rates are per 100,000

**Table 2:** Fixed effects spatial autoregressive models predicting opioid overdose rates by arrests for possession of heroin in Chicago, 2016 - 2019, *N*=308

	EMS-Response Opioid Overdose Rate						Fatal Opioid Overdose Rate					
	Model 1		Model 2		Model 3		Model 1		Model 2		Model 3	
	b	SE	b	SE	b	SE	b	SE	b	SE	b	SE
Heroin Possession Arrest Rate												
Local Arrests	0.360***	(0.08)	0.318***	(0.08)	0.359***	(0.08)	−0.142	(0.08)	−0.124	(0.07)	−0.131	(0.07)
Spatial Spillover of Arrests	1.620***	(0.18)			1.636***	(0.19)	0.582***	(0.17)			0.308	(0.17)
Social Spillover of Arrests			2.409*	(1.00)	−0.256	(0.94)			4.624***	(0.89)	4.002***	(0.95)
Time-Varying Controls												
Homeless Rate	0.0249	(0.02)	0.0284	(0.03)	0.0254	(0.02)	0.0674**	(0.02)	0.0604*	(0.02)	0.0593*	(0.02)
Crime Rate	1.045***	(0.19)	1.056***	(0.22)	1.040***	(0.20)	0.681***	(0.20)	0.778***	(0.20)	0.780***	(0.20)
Year	0.0279	(0.01)	0.0275	(0.02)	0.0299	(0.02)	0.0129	(0.01)	−0.0185	(0.02)	−0.0204	(0.02)
Opioid Overdose Rate Spatial Lag	0.193	(0.10)	0.483***	(0.09)	0.195	(0.10)	0.215*	(0.09)	0.197*	(0.09)	0.183*	(0.09)
Constant	0.246***	(0.01)	0.278***	(0.01)	0.246***	(0.01)	0.258***	(0.01)	0.251***	(0.01)	0.249***	(0.01)
AIC	24.34		90.99		26.26		46.06		32.01		30.91	
BIC	50.45		117.1		56.10		72.17		58.12		60.75	
Log Liklihood	−5.169		−38.50		−5.132		−16.03		−9.004		−7.454	

All measures are standardized to a mean of 0 and standard deviation of 1. * *p* < .05, ** *p* < .01, *** *p* < .001

**Table 3:** Fixed effects spatial autoregressive models predicting opioid overdose rates by arrests for possession of synthetic narcotics in Chicago, 2016 - 2019, *N*=308

	EMS-Response Opioid Overdose Rate						Fatal Opioid Overdose Rate					
	Model 1		Model 2		Model 3		Model 1		Model 2		Model 3	
	b	SE	b	SE	b	SE	b	SE	b	SE	b	SE
Synthetic Possession Arrest Rate												
Local Arrests	0.129***	(0.03)	0.158***	(0.03)	0.134***	(0.03)	0.133***	(0.03)	0.144***	(0.03)	0.135***	(0.03)
Spatial Spillover of Arrests	0.182**	(0.06)			0.212***	(0.06)	0.0561	(0.06)			0.0719	(0.06)
Social Spillover of Arrests			−0.167	(0.26)	−0.439	(0.27)			−0.127	(0.24)	−0.220	(0.25)
Time-Varying Controls												
Homeless Rate	0.00556	(0.03)	0.000552	(0.03)	0.00639	(0.03)	0.0519*	(0.02)	0.0500*	(0.02)	0.0525*	(0.02)
Crime Rate	0.999***	(0.21)	0.974***	(0.21)	0.961***	(0.21)	0.407*	(0.19)	0.390*	(0.20)	0.390*	(0.20)
Year	0.0136	(0.02)	0.0440*	(0.02)	0.0370	(0.02)	−0.00743	(0.02)	0.00735	(0.02)	0.00378	(0.02)
Opioid Overdose Rate Spatial Lag	0.437***	(0.09)	0.509***	(0.08)	0.417***	(0.09)	0.141	(0.10)	0.174	(0.09)	0.127	(0.10)
Constant	0.268***	(0.01)	0.271***	(0.01)	0.267***	(0.01)	0.250***	(0.01)	0.250***	(0.01)	0.250***	(0.01)
AIC	70.80		79.76		70.13		30.39		31.10		31.63	
BIC	96.92		105.9		99.97		56.50		57.22		61.47	
Log Liklihood	−28.40		−32.88		−27.06		−8.197		−8.552		−7.816	

All measures are standardized to a mean of 0 and standard deviation of 1. * *p* < .05, ** *p* < .01, *** *p* < .001

**Table 4:** Fixed effects spatial autoregressive models predicting opioid overdose rates by arrests for manufacturing/distribution of heroin in Chicago, 2016 - 2019, *N*=308

	EMS-Response Opioid Overdose Rate						Fatal Opioid Overdose Rate					
	Model 1		Model 2		Model 3		Model 1		Model 2		Model 3	
	b	SE	b	SE	b	SE	b	SE	b	SE	b	SE
Heroin Manufacturing/Distribution Arrest Rate												
Local Arrests	0.669***	(0.06)	0.645***	(0.05)	0.659***	(0.05)	−0.0551	(0.06)	−0.0413	(0.06)	−0.0446	(0.06)
Spatial Spillover of Arrests	0.599***	(0.17)			0.600***	(0.16)	0.184	(0.17)			0.208	(0.17)
Social Spillover of Arrests			−3.232**	(1.04)	−3.254**	(1.03)			1.958	(1.22)	2.084	(1.22)
Time-Varying Controls												
Homeless Rate	0.0709***	(0.02)	0.0682**	(0.02)	0.0667**	(0.02)	0.0701**	(0.03)	0.0750**	(0.03)	0.0733**	(0.03)
Crime Rate	0.477**	(0.18)	0.444*	(0.18)	0.467**	(0.18)	0.636**	(0.22)	0.624**	(0.21)	0.641**	(0.21)
Year	0.0372**	(0.01)	0.0374**	(0.01)	0.0404**	(0.01)	0.0232	(0.01)	0.0257	(0.01)	0.0228	(0.01)
Opioid Overdose Rate Spatial Lag	0.389***	(0.09)	0.506***	(0.07)	0.350***	(0.09)	0.233*	(0.09)	0.238*	(0.09)	0.232*	(0.09)
Constant	0.223***	(0.01)	0.221***	(0.01)	0.219***	(0.01)	0.265***	(0.01)	0.264***	(0.01)	0.263***	(0.01)
AIC	−16.57		−12.94		−24.41		58.83		57.47		57.96	
BIC	9.540		13.17		5.430		84.94		83.58		87.80	
Log Liklihood	15.29		13.47		20.21		−22.42		−21.74		−20.98	

All measures are standardized to a mean of 0 and standard deviation of 1. * *p* < .05, ** *p* < .01, *** *p* < .001

**Table 5:** Fixed effects spatial autoregressive models predicting opioid overdose rates by arrests for manufacturing/distribution of synthetic narcotics in Chicago, 2016 - 2019, *N*=308

	EMS-Response Opioid Overdose Rate						Fatal Opioid Overdose Rate					
	Model 1		Model 2		Model 3		Model 1		Model 2		Model 3	
	b	SE	b	SE	b	SE	b	SE	b	SE	b	SE
Synthetic Manufacturing/Distribution Arrest Rate												
Local Arrests	−0.116***	(0.03)	−0.118***	(0.03)	−0.118***	(0.03)	−0.0400	(0.03)	−0.0437	(0.03)	−0.0450	(0.03)
Spatial Spillover of Arrests	0.0142	(0.08)			0.000403	(0.08)	−0.0272	(0.08)			−0.0573	(0.08)
Social Spillover of Arrests			0.582	(0.44)	0.582	(0.44)			1.213**	(0.43)	1.257**	(0.43)
Time-Varying Controls												
Homeless Rate	0.0244	(0.03)	0.0228	(0.03)	0.0228	(0.03)	0.0735**	(0.03)	0.0721**	(0.02)	0.0710**	(0.02)
Crime Rate	1.214***	(0.21)	1.234***	(0.21)	1.234***	(0.21)	0.587**	(0.20)	0.620**	(0.20)	0.638**	(0.20)
Year	0.0556***	(0.02)	0.0319	(0.02)	0.0319	(0.02)	0.0253	(0.01)	−0.0266	(0.02)	−0.0270	(0.02)
Opioid Overdose Rate Spatial Lag	0.606***	(0.07)	0.590***	(0.07)	0.590***	(0.08)	0.232*	(0.09)	0.165	(0.10)	0.160	(0.10)
Constant	0.274***	(0.01)	0.274***	(0.01)	0.274***	(0.01)	0.265***	(0.01)	0.261***	(0.01)	0.261***	(0.01)
AIC	93.56		91.81		93.81		58.46		50.55		51.98	
BIC	119.7		117.9		123.7		84.57		76.66		81.82	
Log Liklihood	−39.78		−38.91		−38.91		−22.23		−18.27		−17.99	

All measures are standardized to a mean of 0 and standard deviation of 1. * *p* < .05, ** *p* < .01, *** *p* < .001

## Footnotes

1 See the descriptives presented in Table 1.

2
https://www.cookcountyil.gov/news/cook-county-medical-examiners-office-confirms-record-2000-opioid-overdose-deaths-cook-county

3
https://www.cookcountyil.gov/news/cook-county-medical-examiners-office-registers-record-number-gunrelated-homicides-2020

4 While naloxone access laws have faced criticism due to concerns that availability might encourage riskier opioid use, and several studies have demonstrated an indirect positive effect of naloxone laws on fatal opioid overdose rates (Erfanian et al. 2019; Doleac and Mukherjee 2018), Doleac and Mukherjee (2022) still note the harm reduction potential of naloxone when accompanied by additional harm-reduction programs.

5
https://data.cityofchicago.org/Public-Safety/Crimes-2001-to-Present/ijzp-q8t2/about_data

6 The 2010–2014 ACS data was used due to methodological constraints for small-area estimation. We deliberately avoided using 2015–2019 ACS data to prevent temporal overlap with our outcome period, as this would incorporate predictor data collected after our study began. This approach provides consistent denominators across all study years.

7 While the Point-in-Time (PIT) count cannot provide an exact number of people experiencing homelessness in the city of Chicago, it utilizes data obtained from volunteers, such as city employees and homeless shelter providers, who spend an evening canvassing the city every two years. This data is used to provide a yearly estimate of homelessness in Chicago.

8 Appendix Table A2 presents the yearly global Moran’s I coefficients for each dependent variable.

9 Appendix Table A3 presents the Lagrange Multiplier postestimation tests suggesting a spatial lag model is an appropriate modeling strategy for the data.

10 Appendix Table A4 presents results from the Hausman postestimation tests suggesting fixed effects are appropriate for the analysis.

11 While equivalent in this study, we retain separate notations for the W and M matrices, consistent with the literature, as in principle, they may or may not be equivalent (Lee and Yu 2010).

12 We use the language of “effects” based on theoretical expectations and our use of fixed “effects” models.Still, short of a randomized design, caution is needed around a strict causal interpretation.

13 We thank a reviewer for this comment.

## References

[R1] Allison PD (2009) Fixed Effects Regression Models. Quantitative Applications in the Social Sciences 160. Sage, Los Angeles

[R2] Anselin L (2000) Computing environments for spatial data analysis. J Geogr Syst 2:201–220. 10.1007/PL00011455

[R3] Anselin L (2003) Spatial Externalities, Spatial Multipliers, And Spatial Econometrics. Int Reg Sci Rev 26(2):153–166. 10.1177/0160017602250972

[R4] Anselin L, Bera AK, Florax R, Yoon MJ (1996) Simple diagnostic tests for spatial dependence. Reg Sci Urban Econ 26(1):77–104. 10.1016/0166-0462(95)02111-6

[R5] Baller RD, Anselin L, Messner SF, Deane G, Hawkins DF (2001) Structural covariates of US county homicide rates: Incorporating spatial effects. Criminology 39(3):561–588. 10.1111/j.1745-9125.2001.tb00933.x

[R6] Bennett AS, Scheidell J, Bowles JM, Khan M, Roth A, Hoff L, Marini C, Elliott L (2022) Naloxone protection, social support, network characteristics, and overdose experiences among a cohort of people who use illicit opioids in New York City. Harm Reduct J 19(1):20. 10.1186/s12954-022-00604-w 35246165 PMC8894821

[R7] Bivens KM (2019) Reducing harm by designing discourse and digital tools for opioid users’ contexts: the Chicago Recovery Alliance’s community-based context of use and PwrdBy’s technology-based context of use. Commun Design Quart Rev 7(2):17–27. 10.1145/3358931.3358935

[R8] Bohnert AS, Valenstein M, Bair MJ, Ganoczy D, McCarthy JF, Ilgen MA, Blow FC (2011a) Association between opioid prescribing patterns and opioid overdose-related deaths. JAMA 305(13):1315–1321. 10.1001/jama.2011.370 21467284

[R9] Bohnert AS, Nandi A, Tracy M, Cerdá M, Tardiff KJ, Vlahov D, Galea S (2011b) Policing and risk of overdose mortality in urban neighborhoods. Drug Alcohol Depend 113(1):62–68. 10.1016/j.drugalcdep.2010.07.008 20727684 PMC3008306

[R10] Boivin R, D’Elia M (2017) A network of neighborhoods: Predicting crime trips in a large Canadian city. J Res Crime Delinq 54(6):824–846. 10.1177/0022427817705935

[R11] Boivin R, Felson M (2018) Crimes by visitors versus crimes by residents: the influence of visitor inflows. J Quant Criminol 34:465–480. 10.1007/s10940-017-9341-1

[R12] Bouchard M, Hashimi S, Tsai K, Lampkin H, Jozaghi E (2018) Back to the core: a network approach to bolster harm reduction among persons who inject drugs. Int J Drug Pol 51:95–104. 10.1016/j.drugpo.2017.10.006 29227844

[R13] Braga AA, Papachristos AV, Hureau DM (2017) The effects of hot spots policing on crime: An updated systematic review and meta-analysis. Justice Q 31(4):633–663. 10.1080/07418825.2012.673632

[R14] Browning CR, Calder CA, Soller B, Jackson AL, Dirlam J (2017) Ecological networks and neighborhood social organization. Am J Sociol 122(6):1939–1988. 10.1086/691261 PMC5786432 29379218

[R15] Federal Bureau of Investigation (FBI): Uniform Crime Reporting System (UCR) (2019) Offense Definitions. Criminal Justice Information Services Division. https://ucr.fbi.gov/crime-in-the-u.s/2019/crime-in-the-u.s.-2019/topic-pages/offense-definitions

[R16] Byles H, Sedaghat N, Rider N, Rioux W, Loverock A, Seo B, Dhanoa A, Orr T, Dunnewold N, Tjosvold L (2024) Barriers to calling emergency services amongst people who use substances in the event of overdose: a scoping review. Int J Drug Policy 132:104559. 10.1016/j.drugpo.2024.104559 39197374

[R17] Carroll J, Green T, Noonan R (2018) Evidence-Based Strategies for Preventing Opioid Overdose: What’s Working in the United States. Centers for Disease Control and Prevention 1–36. https://stacks.cdc.gov/view/cdc/59393/cdc_59393_DS1.pdf

[R18] Case A, Deaton A (2015) Rising morbidity and mortality in midlife among white non-Hispanic Americans in the 21st century. Proc Natl Acad Sci 112(49):15078–15083. 10.1073/pnas.1518393112 26575631 PMC4679063

[R19] Caulkins JP, Gould A, Pardo B, Reuter P, Stein BD (2021) Opioids and the criminal justice system: new challenges posed by the modern opioid epidemic. Annual Review of Criminology 4(1):353–375. 10.1146/annurev-criminol-061020-125715

[R20] Center for Disease Control (CDC) (2024) Understanding the Opioid Overdose Epidemic. CDC Overdose Prevention. https://www.cdc.gov/overdose-prevention/about/understanding-the-opioid-overdose-epidemic.html

[R21] Chalfin A, McCrary J (2017) Criminal deterrence: A review of the literature. J Econ Lit 55(1):5–48. 10.1257/jel.20141147

[R22] Chaney RA, Rojas-Guyler L (2015) Spatial patterns of adolescent drug use. Appl Geogr 56:71–82. 10.1016/j.apgeog.2014.11.002

[R23] Chicago Department of Public Health (CDPH) (2021) Health Alert: Chicago Opioid Update. Chicago Department of Public Health. City of Chicago. May 14. https://www.chicago.gov/city/en/depts/cdph/supp_info/behavioral-health/opioid-use1.html

[R24] Chicago Department of Public Health (CDPH) (2024) Chicago Department of Public Health Announces Strategy to Combat Opioid Overdoses. https://www.chicago.gov/city/en/depts/cdph/provdrs/health_protection_and_response/news/2024/may/cdph-press-release-05-15-2024.html

[R25] City of Chicago (2024) Point in Time (PIT) Count. Family and Support Services. https://www.chicago.gov/city/en/depts/fss/provdrs/emerg/svcs/PITcount.html

[R26] Clark AK, Wilder CM, Winstanley EL (2014) A systematic review of community opioid overdose prevention and naloxone distribution programs. J Addict Med 8(3):153–163. 10.1097/ADM.0000000000000034 24874759

[R27] Cooper H, Moore L, Gruskin S, Krieger N (2005) The impact of a police drug crackdown on drug injectors’ ability to practice harm reduction: a qualitative study. Soc Sci Med 61(3):673–684. 10.1016/j.socscimed.2004.12.030 15899325

[R28] Cooper LA, Roter DL, Carson KA, Beach MC, Sabin JA, Greenwald AG, Inui TS (2012) The associations of clinicians’ implicit attitudes about race with medical visit communication and patient ratings of interpersonal care. Am J Public Health 102(5):979–987. 10.2105/AJPH.2011.300558 22420787 PMC3483913

[R29] Cristiano N (2022) Fentanyl contamination as a risk priority: the impact of the fentanyl epidemic on club drug-using behaviours. Subst Use Misuse 57(6):975–982. 10.1080/10826084.2022.2058705 35354372

[R30] De P, Cox J, Boivin JF, Platt RW, Jolly AM (2007) The importance of social networks in their association to drug equipment sharing among injection drug users: a review. Addiction 102(11):1730–1739. 10.1111/j.1360-0443.2007.01936.x 17935581

[R31] Doleac J, Mukherjee A (2018) The moral hazard of lifesaving innovations: naloxone access, opioid abuse, and crime. IZA Discussion Papers 11489. https://hdl.handle.net/10419/180507

[R32] Doleac J, Mukherjee A (2022) The effects of naloxone access laws on opioid abuse, mortality, and crime. The Journal of Law and Economics 65(2):211–238. 10.1086/719588

[R33] Donnelly EA, Wagner J, Stenger M, Cortina HG, O’Connell DJ, Anderson TL (2021) Opioids, race, and drug enforcement: Exploring local relationships between neighborhood context and Black-White opioid-related possession arrests. Crim Justice Policy Rev 32(3):219–244. 10.1177/0887403420911415

[R34] Donnelly EA, Wagner J, Anderson TL, O’Connell D (2022) Revisiting neighborhood context and racial disparities in drug arrests under the opioid epidemic. Race and Justice 12(2):322–343. 10.1177/2153368719877222

[R35] Eggins E, Hine L, Higginson A, Mazerolle L (2020) The impact of arrest and seizure on drug crime and harms: A systematic review. Trends and Issues in Crime and Criminal Justice 602:1–16. 10.3316/INFORMIT.446613706714248

[R36] Elhorst JP (2014) Spatial econometrics: from cross-sectional data to spatial panels. Springer, Berlin, Heidelberg. 10.1007/978-3-642-40340-8

[R37] Enteen L, Bauer J, McLean R, Wheeler E, Huriaux E, Kral AH, Bamberger JD (2010) Overdose prevention and naloxone prescription for opioid users in San Francisco. J Urban Health 87:931–941. 10.1007/s11524-010-9495-8 20967505 PMC3005091

[R38] Erfanian E, Grossman D, Collins AR (2019) The impact of naloxone access laws on opioid overdose deaths in the US. Rev Reg Stud 49(1):45–72

[R39] Eswaran V, Allen KC, Cruz DS, Lank PM, McCarthy DM, Kim HS (2020) Development of a take-home naloxone program at an urban academic emergency department. J Am Pharm Assoc 60(6):e324–e331. 10.1016/j.japh.2020.06.017 32690447

[R40] Evans M, Graif C, Matthews SA (2023) The role of infant health problems in constraining interneighborhood mobility: implications for citywide employment networks. J Health Soc Behav 64(4):555–577. 10.1177/00221465231172176 37272013 PMC10683334

[R41] Felson M, Boivin R (2015) Daily crime flows within a city. Crime Sci 4:1–10. 10.1186/s40163-015-0039-0

[R42] Friedman J, Shover CL (2023) Charting the fourth wave: Geographic, temporal, race/ethnicity and demographic trends in polysubstance fentanyl overdose deaths in the United States, 2010–2021. Addiction 118(12):2477–2485. 10.1111/add.16318 37705148

[R43] Gershowitz AM (2020) The Opioid Doctors: Is Losing Your License a Sufficient Penalty for Dealing Drugs? Hastings LJ 72:871. https://heinonline.org/HOL/Page?collection=journals&handle=hein.journals/hastlj72&id=872&men_tab=srchresults

[R44] Giftos J, Tesema L (2018) When less is more: reforming the criminal justice response to the opioid epidemic. Judges J 57:28. https://heinonline.org/HOL/Page?handle=hein.journals/judgej57&div=11&g_sent=1&casa_token=&collection=journals

[R45] Graham MR, Kutzbach MJ, McKenzie B (2014) Design comparison of LODES and ACS commuting data products. Center for Economic Studies, U.S. Census Bureau. No. 14–38. https://ideas.repec.org/p/cen/wpaper/14-38.html

[R46] Graif C, Gladfelter AS, Matthews SA (2014) Urban poverty and neighborhood effects on crime: incorporating spatial and network perspectives. Sociol Compass 8(9):1140–1155. 10.1111/soc4.12199 27375773 PMC4928692

[R47] Graif C, Lungeanu A, Yetter AM (2017) Neighborhood isolation in chicago: violent crime effects on structural isolation and homophily in inter-neighborhood commuting networks. Social Networks 51:40–59. 10.1016/j.socnet.2017.01.007 29104357 PMC5663310

[R48] Graif C, Freelin BN, Kuo YH, Wang H, Li Z, Kifer D (2021) Network spillovers and neighborhood crime: a computational statistics analysis of employment-based networks of neighborhoods. Justice Q 38(2):344–374. 10.1080/07418825.2019.1602160 34025017 PMC8132726

[R49] Harper S, Riddell CA, King NB (2021) Declining life expectancy in the United States: missing the trees for the forest. Annu Rev Public Health 42(1):381–403. 10.1146/annurev-publhealth-082619-104231 33326297

[R50] Hawk KF, Vaca FE, D’Onofrio G (2015) Focus: Addiction: Reducing fatal opioid overdose: Prevention, treatment and harm reduction strategies. The Yale journal of biology and medicine 88(3):235. https://pmc.ncbi.nlm.nih.gov/articles/PMC4553643/26339206 PMC4553643

[R51] Hipp JR, Williams SA (2020) Advances in spatial criminology: The spatial scale of crime. Ann Rev Criminol 3(1):75–95. 10.1146/annurev-criminol-011419-041423

[R52] Hipp JR, Faris RW, Boessen A (2012) Measuring ‘neighborhood’: Constructing network neighborhoods. Social Networks 34(1):128–140. 10.1016/j.socnet.2011.05.002

[R53] Holland A, Stevens A, Harris M, Lewer D, Sumnall H, Stewart D, Gilvarry E (2023) Analysis of the UK Government’s 10-Year Drugs Strategy—a resource for practitioners and policymakers. J Public Health 45(2):e215–e224. 10.1093/pubmed/fdac114 PMC10273368 36309802

[R54] Hopwood T, Dowd-Green C, Mason M, Stewart RW (2020) Unintentional use of fentanyl attributed to surreptitious cannabis adulteration. J Am Pharm Assoc 60(6):e370–e374. 10.1016/j.japh.2020.07.003 32778518

[R55] Johnson NJ, Roman CG, Mendlein AK, Harding C, Francis M, Hendrick L (2020) Exploring the influence of drug trafficking gangs on overdose deaths in the largest narcotics market in the eastern United States. Social Sciences 9(11):202. 10.3390/socsci9110202

[R56] Kelling C, Graif C, Korkmaz G, Haran M (2021) Modeling the social and spatial proximity of crime: domestic and sexual violence across neighborhoods. J Quant Criminol 37:481–516. 10.1007/s10940-020-09454-w 34149156 PMC8210633

[R57] Kirk DS, Wakefield S (2018) Collateral consequences of punishment: A critical review and path forward. Ann Rev Criminol 1(1):171–194. 10.1146/annurev-criminol-032317-092045

[R58] Knoebel RW, Kim SJ (2023) Impact of Covid-19 pandemic, social vulnerability, and opioid overdoses. Chicago AJPM Focus 2(2):100086. 10.1016/j.focus.2023.100086 36789246 PMC9911148

[R59] Kopak AM, Lawson SW, Hoffmann NG (2018) Criminal justice contact and relapse among patients seeking treatment for opioid use disorder. J Drug Issues 48(1):134–147. 10.1177/0022042617740911

[R60] Krawczyk N, Schneider KE, Eisenberg MD, Richards TM, Ferris L, Mojtabai R, Stuart EA, Casey Lyons B, Jackson K, Weiner JP, Saloner B (2020) Opioid overdose death following criminal justice involvement: Linking statewide corrections and hospital databases to detect individuals at highest risk. Drug Alcohol Depend 213:107997. 10.1016/j.drugalcdep.2020.107997 32534407

[R61] Kwan MP, Peterson RD, Browning CR, Burrington LA, Calder CA, Krivo LJ (2008) Reconceptualizing Sociogeographic Context for the Study of Drug Use, Abuse, and Addiction. Geography and drug addiction. Springer, Netherlands, Dordrecht, pp 437–446. 10.1007/978-1-4020-8509-3_26

[R62] Lankenau SE, Wagner KD, Silva K, Kecojevic A, Iverson E, McNeely M, Kral AH (2013) Injection drug users trained by overdose prevention programs: responses to witnessed overdoses. J Community Health 38:133–141. 10.1007/s10900-012-9591-7 22847602 PMC3516627

[R63] Lee LF, Yu J (2010) Estimation of spatial autoregressive panel data models with fixed effects. J Econ 154(2):165–185. 10.1016/j.jeconom.2009.08.001

[R64] Levy BL, Phillips NE, Sampson RJ (2020) Triple disadvantage: neighborhood networks of everyday urban mobility and violence in US cities. Am Sociol Rev 85(6):925–956. 10.1177/0003122420972323

[R65] Lim S, Seligson AL, Parvez FM, Luther CW, Mavinkurve MP, Binswanger IA, Kerker BD (2012) Risks of drug-related death, suicide, and homicide during the immediate post-release period among people released from New York City jails, 2001–2005. Am J Epidemiol 175(6):519–526. 10.1093/aje/kwr327 22331462 PMC7159090

[R66] Lister JJ, Weaver A, Ellis JD, Himle JA, Ledgerwood DM (2020) A systematic review of rural-specific barriers to medication treatment for opioid use disorder in the United States. Am J Drug Alcohol Abuse 46(3):273–288. 10.1080/00952990.2019.1694536 31809217

[R67] Mason M, Cheung I, Walker L (2004) Substance use, social networks, and the geography of urban adolescents. Subst Use Misuse 39(10–12):1751–1777. 10.1081/JA-200033222 15587950

[R68] Matthews SA, Yang TC (2013) Spatial polygamy and contextual exposures (spaces) promoting activity space approaches in research on place and health. Am Behav Sci 57(8):1057–1081. 10.1177/0002764213487345 24707055 PMC3975622

[R69] Maxwell S, Bigg D, Stanczykiewicz K, Carlberg-Racich S (2006) Prescribing naloxone to actively injecting heroin users: a program to reduce heroin overdose deaths. J Addict Dis 25(3):89–96. 10.1300/J069v25n03_11 16956873

[R70] Mazerolle L, Soule D, Rombouts S (2007) Drug law enforcement: A review of the evaluation literature. Police Q 10(2):115–153. 10.1177/1098611106287776

[R71] Mazerolle L, Eggins E, Higginson A (2020) Street-level drug law enforcement: An updated systematic review. Trends and issues in crime and criminal justice 599:1–20. 10.3316/informit.379385946414662

[R72] McDonald R, Eide D, Skurtveit S, Clausen T (2024) Pills and the damage done: the opioid epidemic as man-made crisis. Front Public Health 11(1241404):1241404. 10.3389/fpubh.2023.1241404 38283292 PMC10820717

[R73] Mears DP, Bhati AS (2006) No community is an island: The effects of resource deprivation on urban violence in spatially and socially proximate communities. Criminology 44(3):509–548. 10.1111/j.1745-9125.2006.00056.x

[R74] Mercer F, Miler JA, Pauly B, Carver H, Hnízdilová K, Foster R, Parkes T (2021) Peer support and overdose prevention responses: a systematic ‘state-of-the-art’ review. Int J Environ Res Public Health 18(22):12073. 10.3390/ijerph182212073 34831839 PMC8621858

[R75] Messmer SE, Elmes AT, Jimenez AD, Murphy AL, Guzman M, Watson DP, Poorman E, Mayer S, Infante AF, Keller EG, Whitfield K, Jarrett JB (2023) Outcomes of a mobile medical unit for low-threshold buprenorphine access targeting opioid overdose hot spots in Chicago. J Substance Use Addict Treatment 150:209054. 10.1016/j.josat.2023.209054 PMC10330226 37088399

[R76] Morenoff JD, Sampson RJ, Raudenbush SW (2001) Neighborhood inequality, collective efficacy, and the spatial dynamics of urban violence. Criminology 39(3):517–558. 10.1111/j.1745-9125.2001.tb00932.x

[R77] Morrison CN, Rundle AG, Branas CC, Chihuri S, Mehranbod C, Li G (2020) The unknown denominator problem in population studies of disease frequency. Spatial Spatio-Temp Epidemiol 35:100361. 10.1016/j.sste.2020.100361 PMC7609977 33138954

[R78] Nagin DS (2013) Deterrence in the twenty-first century. Crime Justice 42(1):199–263. 10.1086/670398

[R79] Newmyer L, Evans M, Graif C (2022) Socially connected neighborhoods and the spread of sexually transmitted infections. Demography 59(4):1299–1323. 10.1215/00703370-10054898 35838157 PMC9707946

[R80] Nguyen H, Loughran TA, Topalli V (2023) Crime, consumption, and choice: On the interchangeability of licit and illicit income. J Res Crime Delinq 60(4):416–454. 10.1177/00224278231152624

[R81] Novick P (2019) Healers Or Dealers: The Effect of Doctors Committing Health Care Fraud on the Opioid Epidemic. Int’l Comp., Policy & Ethics L. Rev 3:453. https://heinonline.org/HOL/Page?handle=hein.journals/icpelr3&div=18&g_sent=1&casa_token=&collection=journals

[R82] Ostrach B, Potter R, Wilson CG, Carpenter D (2022) Ensuring buprenorphine access in rural community pharmacies to prevent overdoses. J Am Pharm Assoc 62(2):588–597. 10.1016/j.japh.2021.10.002 34674965

[R83] Papachristos G, Sofianos A, Adamides E (2013) System interactions in socio-technical transitions: Extending the multi-level perspective. Environ Innov Soc Trans 7:53–69. 10.1016/j.eist.2013.03.002

[R84] Papachristos AV, Bastomski S (2018) Connected in crime: the enduring effect of neighborhood networks on the spatial patterning of violence. American Journal of Sociology 124(2):517–568. 10.1086/699217

[R85] Pichini S, Solimini R, Berretta P, Pacifici R, Busardò FP (2018) Acute intoxications and fatalities from illicit fentanyl and analogues: an update. Ther Drug Monit 40(1):38–51. 10.1097/FTD.0000000000000465 29120973

[R86] Ray B, Korzeniewski SJ, Mohler G, Carroll JJ, Del Pozo B, Victor G, Huynh P, Hedden BJ (2023) Spatiotemporal analysis exploring the effect of law enforcement drug market disruptions on overdose, Indianapolis, Indiana, 2020–2021. Am J Public Health 113(7):750–758. 10.2105/AJPH.2023.30729110.2105/AJPH.2023.307291 37285563 PMC10262257

[R87] Reuter PH, MacCoun RJ (1992) Street drug markets in inner-city neighbourhoods. Urban America, Rand, Santa Monica, California, 227–251. https://files.eric.ed.gov/fulltext/ED361439.pdf#page=230

[R88] Rushovich T, Arwady MA, Salisbury-Afshar E, Arunkumar P, Aks S, Prachand N (2022) Opioid-related overdose deaths by race and neighborhood economic hardship in Chicago. J Ethn Subst Abuse 21(1):22–35. 10.1080/15332640.2019.1704335 31990245

[R89] Sampson RJ (1986) Crime in cities: The effects of formal and informal social control. Crime Justice 8:271–311. 10.1086/449125

[R90] Sampson RJ (2012) Great American city: Chicago and the enduring neighborhood effect. University of Chicago Press

[R91] Sampson RJ, Morenoff JD, Gannon-Rowley T (2002) Assessing “neighborhood effects”: Social processes and new directions in research. Ann Rev Sociol 28(1):443–478. 10.1146/annurev.soc.28.110601.141114

[R92] Sampson RJ, Morenoff JD (2004) Spatial (dis) advantage and homicide in Chicago neighborhoods. In: Spatially integrated social science, Oxford University Press, pp 145–170

[R93] Schaefer DR (2012) Youth co-offending networks: An investigation of social and spatial effects. Social Networks 34(1):141–149. 10.1016/j.socnet.2011.02.001

[R94] Schumann H, Erickson T, Thompson TM, Zautcke JL, Denton JS (2008) Fentanyl epidemic in Chicago, Illinois and surrounding cook county. Clin Toxicol 46(6):501–506. 10.1080/15563650701877374 18584361

[R95] Seto CH, Graif C, Khademi A, Honavar VG, Kelling CE (2022) Connected in health: Place-to-place commuting networks and Covid-19 spillovers. Health Place 77:102891. 10.1016/j.healthplace.2022.102891 35970068 PMC9365871

[R96] StataCorp (2019) Spatial autoregressive models for panel data. https://www.stata.com/manuals/spspxtregress.pdf

[R97] Taylor S (2015) Crime and criminality: A multidisciplinary approach. Routledge. 10.4324/9781315713298

[R98] Telep CW, Weisburd D, Gill CE, Vitter Z, Teichman D (2014) Displacement of crime and diffusion of crime control benefits in large-scale geographic areas: A systematic review. J Exp Criminol 10:515–548. 10.1007/s11292-014-9208-5

[R99] Tucker JA, Stoutenborough JW, Beverlin RM (2012) Geographic proximity in the diffusion of concealed weapons permit laws. Politics & Policy 40(6):1081–1105. 10.1111/j.1747-1346.2012.00399.x

[R100] Victor GA, Bailey K, Ray B (2021) Buprenorphine treatment intake and critical encounters following a nonfatal opioid overdose. Subst Use Misuse 56(7):988–996. 10.1080/10826084.2021.1901933 33749520

[R101] Wang H, Kifer D, Graif C, Li Z (2016) Crime rate inference with big data. In Proceedings of the 22nd ACM SIGKDD international conference on knowledge discovery and data mining, pp. 635–644. 10.1145/2939672.2939736

[R102] Weisburd D, Telep CW (2013) Spatial displacement and diffusion of crime control benefits revisited: New evidence on why crime doesn’t just move around the corner. In: Tilley N, Farrell G (Eds) The reasoning criminologist: Essays in honour of Ronald V. Clarke, Routledge, pp 142–159

[R103] Wood E, Spittal PM, Small W, Kerr T, Li K, Hogg RS, Tyndall MW, Montaner JS, Schechter MT (2004) Displacement of Canada’s largest public illicit drug market in response to a police crackdown. CMAJ 170(10):1551–1556. 10.1503/cmaj.103192810.1503/cmaj.1031928 15136548 PMC400719

[R104] Zhang A, Balles JA, Nyland JE, Nguyen TH, White VM, Zgierska AE (2022) The relationship between police contacts for drug use-related crime and future arrests, incarceration, and overdoses: a retrospective observational study highlighting the need to break the vicious cycle. Harm Reduct J 19(1):67. 10.1186/s12954-022-00652-2 35761290 PMC9238075

